# IT’S BEYOND PERSONAL: MULTILEVEL FACTORS AFFECTING THE WILLINGNESS FOR PALLIATIVE CARE UTILIZATION

**DOI:** 10.1093/geroni/igae098.0212

**Published:** 2024-12-31

**Authors:** Juncheng Tong, Jiawei Cao

**Affiliations:** Nanjing University of Chinese Medicine, Nanjing, Jiangsu, China (People’s Republic); Nanjing University Of Chinese Medicine, Nanjing, Jiangsu, China (People’s Republic)

## Abstract

With so much effort devoted to the development of palliative care, significant progress has been achieved in the past few years in China. One of the foci is the patient education and public advocacy. However, evaluation research on this aspect is scarce, and little is known regarding people’s willingness to engage in conversations about palliative care and potentially use such services if needed. Thus, this survey study aims to examine the public’s willingness of palliative care utilization and associated factors from micro, meso, and macro levels. Ordinal regression analyses were performed to achieve this goal. Among 2,645 adult participants (aged 18-95), approximately 78% either ‘agree’ or ‘strongly agree’ with the statement that they are willing to use palliative care services if they are terminally ill. Regression results showed that at the micro-level (individual), willing to pay out-of-pocket and believing palliative care can improve end-of-life quality were associated with higher odds of willing to use palliative care. Meso-level measurement of family support and macro-level measurement of filial piety were also significant factors. Specifically, more family support was associated with an increase in the odds of willing to use palliative care (OR = 1.040, p = 0.034); and the odds of willing to use palliative care for people who considered palliative care not to be in line with filial piety was 0.014 (p < 0.001) times that of those who did. Married people were also more likely to be willing to use palliative care. Implications for research, policy, and practice are discussed.

